# Takotsubo cardiomyopathy and Brugada syndrome in a patient with a novel loss-of-function variant in the cardiac sodium channel Na_v_1.5

**DOI:** 10.1016/j.hrcr.2022.01.017

**Published:** 2022-02-04

**Authors:** Tanja Charlotte Frederiksen, Kirstine Calloe, Michelle Geryk, Henrik Kjærulf Jensen

**Affiliations:** ∗ Department of Cardiology, Aarhus University Hospital, Aarhus, Denmark; † Department of Clinical Medicine, Aarhus University, Aarhus, Denmark; ‡ Section for Pathobiological Sciences, Department of Veterinary and Animal Sciences, University of Copenhagen, Frederiksberg, Denmark

**Keywords:** Genetic variant, Takotsubo cardiomyopathy, Brugada syndrome, Cardiac sodium channel, Conduction

**Key Teaching Points**

• We found a novel variant in the *SCN5A* gene (p. Arg376Leu (R376L)) in a patient with a coexistence of Brugada syndrome and takotsubo cardiomyopathy.
• Electrophysiological investigations are consistent with a loss of function in the cardiac sodium channel Na_v_1.5.
• This case report highlights the uncertainty that follows the finding of rare genetic variants and coexisting phenotypes.

## Introduction

Takotsubo cardiomyopathy (TTC) is a stress cardiomyopathy characterized by transient left ventricular dysfunction.^1^ TTC typically presents in postmenopausal women and is triggered by physical or emotional stress, most likely owing to an increased sympathetic activity.^1^ Symptoms resemble those of an acute myocardial infarction and the patients often present with various electrocardiogram (ECG) changes proposing an alteration in cardiac repolarization.^2^ Previously, familial clustering of TTC has been described, suggesting a genetic predisposition to TTC.^3^ Rare, genetic variants have been found in patients with TTC, including variants in the *SCN5A* gene encoding the Na_v_1.5 sodium channel.^4^ Variants in the *SCN5A* gene are also associated with Brugada syndrome (BrS), a genetic disorder with a characteristic repolarization pattern on the ECG leading to increased risk of ventricular arrhythmias.^5^ Previously, case reports have described TTC in patients with a Brugada pattern on the ECG (Brugada phenocopy).^6^^,^^7^

In this study, we describe a female patient presenting with TTC and BrS. The patient has a novel variant in the *SCN5A* gene (c1127G>T, p. Arg376Leu). To support pathogenicity of the *SCN5A* variant, we performed in vitro electrophysiological investigations.

## Case report

**Figure 1:**
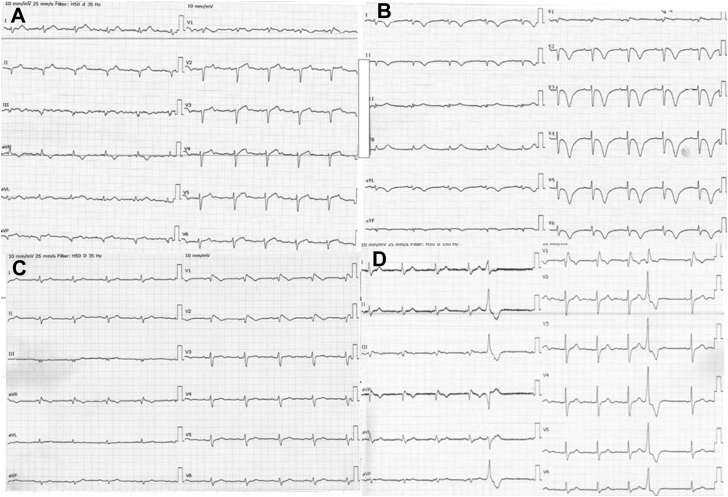
**A:** Electrocardiogram (ECG) at time of admission. PR interval was prolonged (230 ms). Q waves were present in the anterior and inferior leads, as well as poor R wave progression, and there were borderline ST-segment elevations in the precordial leads. **B:** ECG 2 days after the initial presentation. The atrioventricular conduction had normalized (PR interval 185 ms). There were large, inverted T waves in leads I, II, aVL, aVF, and V_2_–V_6_. The QTc interval was prolonged (543 ms corrected with the Fridericia formula). **C:** ECG 3 weeks after discharge. A Brugada type 1 ECG pattern with coved ST-segment elevation >2 mm and inverted T waves was observed in leads V_1_ and V_2_. **D:** ECG 8 years after initial presentation. There was a right bundle branch block. A premature ventricular complex was also observed.

A 70-year-old female patient was admitted to the hospital with chest pain radiating to the left arm. Besides a history of smoking 20 years prior, she had no predispositions to cardiovascular disease and was not taking any medications. The patient had not previously experienced chest pain, shortness of breath, palpitations, or syncope. She had no family history of cardiac disease or sudden cardiac death. The patient had a normal body mass index and was frequently physically active. There was no history of hypertension or hypercholesterolemia. In the ambulance, she was treated with acetylsalicylic acid (ASA) 300 mg and sublingual nitroglycerin, which relieved her pain. At presentation in the emergency department, the patient was free of pain and had stable vital signs. An ECG showed a first-degree atrioventricular block (PR interval 230 ms), Q waves in the anterior and inferior leads, poor R-wave progression, and borderline ST-segment elevations in the precordial leads (Figure 1A). QTc interval was normal. Blood samples showed elevated myocardial necrosis markers (troponin I 1032 ng/L, creatine kinase myocardial band 4.6 μg/L). Total cholesterol was 6.1 mmol/L and low-density lipoprotein cholesterol was 4.3 mmol/L. Echocardiography showed a left ventricle with normal contraction of the basal segments but akinesia and ballooning of the apical segments with a left ventricular ejection fraction (LVEF) of 35%–40%. The patient was treated with heparin 10,000 IU and ticagrelor 180 mg and was taken for an acute coronary angiography. The coronary angiography was normal, with no significant occlusions or stenoses. The patient was admitted for observation and 2 days after the initial presentation an ECG showed normal atrioventricular conduction (PR interval 185 ms) and large, inverted T waves in leads I, II, aVL, aVF, and V_2_–V_6_. The QTc interval was prolonged (543 ms) corrected with the Fridericia formula (Figure 1B). New blood samples showed troponin I reduction to 435 ng/L. When going further into the patient history, the patient revealed that she was feeling emotional stress and sorrow owing to the death of her late spouse, who died 3 months earlier. Based on the patient history, echocardiographic pattern, and normal coronary angiography, a diagnosis of TTC was considered most likely. Although the coronary angiography was normal, it was decided to treat the patient with ASA 75 mg once daily lifelong, ticagrelor 90 mg twice a day for 1 year, and atorvastatin 80 mg once daily lifelong. Furthermore, medical treatment for heart failure was initiated with beta blocker, angiotensin-converting enzyme inhibitor, and an aldosterone antagonist. After 5 days, the patient was discharged in well-being and was seen in the outpatient clinic 3 weeks after the admission. At the outpatient visit, the patient informed the physician that she was doing well and had no cardiovascular symptoms. An echocardiography was performed and LVEF had improved to 40%–45% and with apical hypokinesia of the left ventricle. An ECG was measured, and the large, inverted T waves had now normalized. However, on the new ECG there was coved ST-segment elevation >2 mm and inverted T waves in leads V_1_ and V_2_ (Figure 1C) resembling a Brugada type 1 ECG pattern. The patient was offered genetic testing based on a suspicion of BrS, which she accepted. The patient had 3 adult children, who were also offered clinical and genetic testing, which they accepted. All 3 children had normal ECGs and echocardiography and had no cardiovascular symptoms.

After 5 months, she was seen again in the outpatient clinic. On echocardiography, LVEF had normalized to 60% with no regional wall abnormalities. The patient had no cardiovascular complaints and wished to discontinue her medication. Thus, ticagrelor, the angiotensin-converting enzyme inhibitor, and the aldosterone antagonist were discontinued, while she continued with ASA, beta blocker, and atorvastatin.

The patient was recently seen in the outpatient clinic, 8 years after initial presentation. She had no cardiac symptoms and did not have any cardiac complaints or events in the period since admission. Echocardiography was normal with LVEF 60%. ECG was with right bundle branch block and did not fulfill the criteria for Brugada ECG pattern (Figure 1D). A 48-hour Holter monitoring was performed. The Holter monitoring showed sinus rhythm with 7450 premature ventricular complexes over 24 hours, a few premature supraventricular complexes, and no other arrhythmias.

## Methods

### Site-directed mutagenesis and in vitro electrophysiology

cDNA for human (h) Na_v_1.5 isoform 2 (GenBank acc. no. NM_000335) in pcDNA3 was used. R376L was introduced and verified by sequencing (Eurofins, MGW Operon, and GenScript Biotech). Chinese hamster ovary (CHO-K1) cells were transfected with 2 μg hNa_v_1.5, wild-type (WT) or R376L in pcDNA3 using lipofectamine 3000 (Thermo Fisher Scientific, Waltham, MA). Enhanced green fluorescent protein (0.2 μg) was included for identification of transfected cells. Currents were recorded as previously described.^8^

### Ethics

Written informed consent has been obtained from the patient in this study. The study was approved by the local institutional review board.

## Results

### Genetic analysis

**Figure 2:**
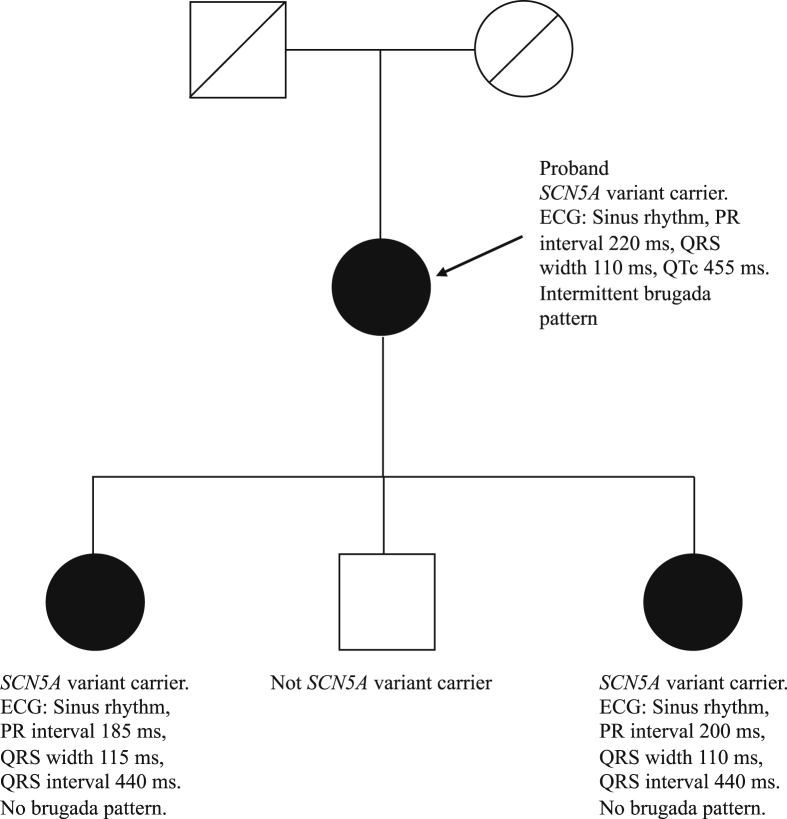
Pedigree. Males are represented by squares and females by circles. A clear symbol shows an unaffected individual, while a black symbol shows an affected individual. Deceased individuals are presented with a diagonal line. The arrow shows the proband. Electrocardiogram (ECG) parameters are presented for affected individuals.

For genetic testing, NimbleGen DNA Capture, Illumina NGS, MLPA, and Sanger sequencing were used. We used a screening panel consisting of 12 genes associated with genetic ion channel cardiac diseases (*CACNA1C, CACNA2D1, CACNB2, GPD1L, KCND3, KCNE1, KCNE2, RANGRF, SCN1B, SCN3B, SCN4B,* and *SCN5A*). A missense variant was found in the *SCN5A* gene (NM_198056.2):c.1127G>T (p. Arg376Leu). The variant could not be found in available databases of human genetic variants and has not been described in the literature, and was thus classified as a variant of uncertain significance (class 3). Two of the patient’s children had the same variant in the *SCN5A* gene, while the third child did not carry the variant. Figure 2 shows a pedigree presenting affected and unaffected family members and ECG parameters for affected individuals.

### Electrophysiological characterization of Na_v_1.5_R376L

**Figure 3:**
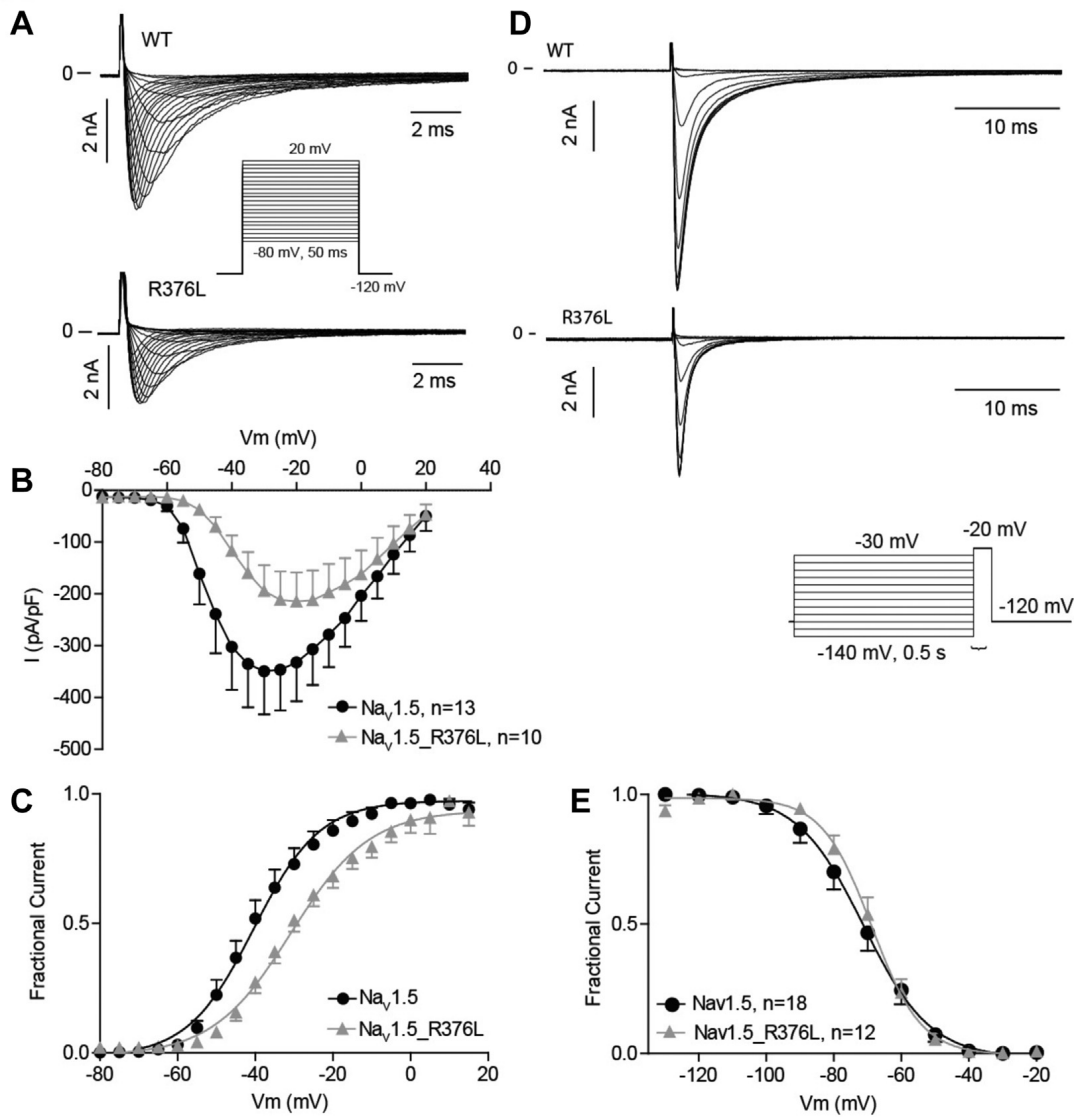
Na_v_1.5 wild-type (WT) or R376L expressed in CHO-K1 cells. **A:** Whole-cell Na_v_1.5 WT and R376L currents. **B:** Peak current density as a function of voltage. **C:** Voltage dependence of activation. **D:** Recordings of steady-state currents. **E:** Voltage dependence of steady-state inactivation.

Peak current density of Na_v_1.5_R376L expressed in CHO-K1 was reduced compared to WT (Figure 3). The voltage dependence of channel activation was shifted, resulting in a half-maximal voltage of activation (V_50_) for WT of -41.0 ± 0.9 mV, n = 18 and -30.8 ± 1.0 mV, n = 12 for R376L (*P* < .0001, unpaired *t* test), consistent with a loss-of-function phenotype. The voltage dependence of steady-state inactivation was also significantly affected, with a half maximal voltage of inactivation (V_50_) -71.2 ± 1.2 mV, n = 18 for WT and -68.8 ± 0.8 mV, n = 12 for R376L (*P* < .0001, unpaired *t* test). Time-dependent recovery from inactivation was not affected.

## Discussion

In this report we present a 70-year-old female patient presenting with TTC. Three weeks after hospital discharge, the patient presented with a Brugada type 1 ECG pattern,^9^ which led to genetic testing. We found a novel variant in the *SCN5A* gene (c.1127G>T, p. Arg376Leu). Electrophysiological studies showed that this variant induces a loss of function in the cardiac sodium channel Na_v_1.5 consistent with Brugada phenocopy.

The underlying pathophysiological mechanisms of TTC are to some extent unknown, but catecholamine surge plays a substantial role.^10^ Since there have been several cases of familial clustering in TTC, a genetic predisposition has also been suggested.^3^ The first genome-wide association study conducted on a cohort of 96 patients with TTC found some potential genetic risk variants, but the study was too small to be conclusive.^11^ Variants in genes associated with various cardiomyopathies have been identified in patients with TTC,^4^ but genetic variants associated with electrical disturbances have also been described. A case report described TTC in a patient with long QT syndrome and a genetic variant in the *KCNH2* gene.^12^

The specific variant we identified in *SCN5A,* R376L, has not been described in the literature, but the *SCN5A* H558R variant has been associated with TTC in a genome-wide association study, suggesting a possible association.^4^ However, the coexistence of TTC and the genetic variant in the *SCN5A* gene likely is coincidental, and we have no data supporting an association between the variant, BrS, and occurrence of TTC. This highlights the uncertainty that follows the interpretation of genetic findings, which should be made clear to the patient by the physician before performing genetic testing.

Variants in the *SCN5A* gene are more commonly associated with sick sinus syndrome, long QT syndrome, atrial fibrillation, and BrS.^13^ BrS is defined by a characteristic ECG pattern with coved-type ST-segment elevation in the right precordial leads (type 1 ECG pattern).^14^ The patients are often young males and might present with clinical signs of BrS, such as family history of sudden cardiac death, symptoms of arrhythmia, documented ventricular arrhythmia, or a positive provocative drug test.^9^ However, the ECG pattern can also occur in patients with other identifiable clinical conditions (Brugada phenocopy), such as metabolic conditions, myocardial disease, pericardial disease, or myocardial ischemia, constituting a diagnostic challenge.^15^ Two case reports described TTC in patients with Brugada phenocopy, where both patients had negative provocative drug tests and no genetic testing was performed.^6^^,^^7^ In our case there is a diagnostic challenge, since the type 1 ECG pattern was identified in the slipstream of TTC and we did not have any ECGs from before the patient was admitted with TTC. Thus, the ECG changes could be a result of possible myocardial alterations in relation to TTC and thereby classified as Brugada phenocopy. However, we found a variant in the *SCN5A* gene, which accounts for approximately 30% of patients diagnosed with BrS,^14^ which we believe is a strong indicator of the diagnosis of BrS in this patient.

## Conclusion

We present a case of a female patient presenting with a coexistence of TTC and BrS. Genetic analysis showed a novel variant in the *SCN5A* gene (p. Arg376Leu (R376L)). Electrophysiological investigations are consistent with a loss-of-function phenotype. The relationship between TTC and variants in the *SCN5A* gene is unclear, and further studies are needed to explore the possible association between rare genetic variants, inherited heart diseases, and TTC.
